# Extracorporeal Shock Wave Therapy for Achilles Tendinopathy

**DOI:** 10.1155/2019/3086910

**Published:** 2019-12-26

**Authors:** Magdalena Stania, Grzegorz Juras, Daria Chmielewska, Anna Polak, Cezary Kucio, Piotr Król

**Affiliations:** ^1^Institute of Sport Sciences, The Jerzy Kukuczka Academy of Physical Education, Mikolowska 72A, 40-065 Katowice, Poland; ^2^Institute of Physiotherapy and Health Sciences, The Jerzy Kukuczka Academy of Physical Education, Mikolowska 72A, 40-065 Katowice, Poland; ^3^Electromyography and Pelvic Floor Muscles Laboratory, The Jerzy Kukuczka Academy of Physical Education, Mikolowska 72A, 40-065 Katowice, Poland; ^4^Rehabilitation Center Technomex, Szparagowa 19, 44-141 Gliwice, Poland; ^5^Department of Internal Disease at the Multispecialty Hospital, Chelmonskiego 28, 43-600 Jaworzno, Poland

## Abstract

Extracorporeal shock wave therapy (ESWT) is among the conservative treatments for Achilles tendinopathy. Unfortunately, no optimal application parameters have been determined that would ensure ESWT effectiveness in this condition. The aim of the paper is to use research reports on ESWT in patients with Achilles tendinopathy to help practising physiotherapists establish the most effective intervention parameters. A search was conducted using the following databases: PubMed, Scopus, EBSCOhost, and Web of Science. The papers were checked for relevant content and were included based on the following criteria: full-text article published in English and including comprehensive description of shock wave application. Twenty-two articles met the inclusion criteria. Most studies on the effectiveness of ESWT for Achilles tendinopathy included in this narrative review were randomized controlled trials. Two case-control studies, a case series study, prospective audit, clinical trial protocol, and a pilot study were also considered. The majority were prospective studies. Only a few authors presented the findings from retrospective observations. The two modalities of shock wave therapy used for Achilles tendinopathy are focused shock waves and radial shock waves. The literature contains reports presenting mainly beneficial effects of ESWT in patients with Achilles tendinopathy.

## 1. Introduction

Järvinen et al. mentioned tendinopathy as being among the most common clinical diagnoses of Achilles disorders (55–65%) [1]. In the Netherlands, the incidence of Achilles tendinopathy is 1.85 per 1,000 Dutch patients registered with general practitioners [2]. Achilles tendinopathy is frequently diagnosed in athletes and physical workers whose activity is associated with major mechanical loading that exceeds the tendon's capacity. Men have a higher prevalence of Achilles tendinopathy compared to premenopausal women, which is probably due to higher levels of physical activity [3]. Also, patients with unilateral Achilles tendinopathy are at high risk of developing contralateral symptoms [4].

Achilles tendinopathy is confirmed by a clinical symptom triad of pain, swelling, and limited function [5]. Achilles tendon injuries are classified by the anatomical area into noninsertional and insertional. The major symptom of noninsertional tendinopathy is pain located 2 to 6 cm proximal to the insertion of the tendon into the calcaneus [6, 7]. Patients suffering from insertional pathology usually present with lesions in the distal portion of the structure, i.e., posterosuperior calcaneal protuberance [8].

The aetiology of Achilles tendinopathy is associated with several intrinsic and extrinsic factors. The intrinsic factors include impaired blood supply, gastrocnemius-soleus dysfunction, age, sex, body weight, metabolic disorders, lateral ankle instability, foot joint hypermobility, and foot deformities. The extrinsic factors that might contribute to Achilles tendinopathy are several sport disciplines (volleyball, basketball, and running), changes in training schedules, training errors, past injuries, inadequate footwear, and unsuitable training surfaces [1, 5, 9–12]. Repetitive tendon strain (3–8%) promotes cumulative microtrauma [1]. When the reparative capacity of the tendon is exceeded, the tendon sheath may become inflamed, resulting in oedema, pain, and/or tendon degeneration [1, 9].

Histologically, tendinopathy is characterized by the absence of inflammatory cells, poor healing, noninflammatory intratendinous collagen degeneration, collagen fibre disorientation and thinning, hypercellularity with high concentrations of glycosaminoglycans and proteoglycans, and neovascularization [9, 12, 13].

Pain and oedema within the Achilles tendon as well as structure stiffness preclude vigorous physical activity, making the patient seek effective treatments. Initial therapies include conservative interventions, e.g., a variety of physical modalities (laser therapy, ultrasound, electrotherapy, and shock waves) and exercises (also eccentric exercise) [14]. The authors of a recently published meta-analysis do not recommend splints and orthoses to patients with Achilles tendinopathy [15]. If the patient does not benefit from conservative treatment, he or she is referred to surgery, which, in the case of insertional tendinopathy, involves tendon debridement via a medial, midline, or lateral approach with variable detachment of the tendon insertion [8]. Those with noninsertional Achilles tendinopathy undergo minimally invasive procedures, e.g., ventral scraping of the tendon or multiple percutaneous longitudinal tenotomise [16]. The initial results seem encouraging.

Extracorporeal shock wave therapy (ESWT) is among the more conservative treatments for Achilles tendinopathy. Unfortunately, no optimal application parameters have been determined that would ensure ESWT effectiveness in this condition. The aim of this paper is to present research reports on the use and efficacy of extracorporeal shock wave therapy in patients with Achilles tendinopathy. We believe this narrative review will help practising physiotherapists establish the most effective intervention parameters.

## 2. Methods

### 2.1. Data Sources and Searches

The aim of the present paper is to describe the research reports, analysing the use and effectiveness of ESWT in patients with Achilles tendinopathy. A search was conducted using the following databases: PubMed, Scopus, EBSCOhost, and Web of Science (the last search was on the 12th of February 2019). Keywords including “Achilles tendinopathy,” “shock wave therapy,” “extracorporeal shock wave,” “focused extracorporeal shock wave,” “radial extracorporeal shock wave therapy,” “Achilles tendon,” and “treatment” were used in various configurations. Reference lists of all the retrieved articles were manually checked for additional studies.

### 2.2. Study Selection

**Figure 1:**
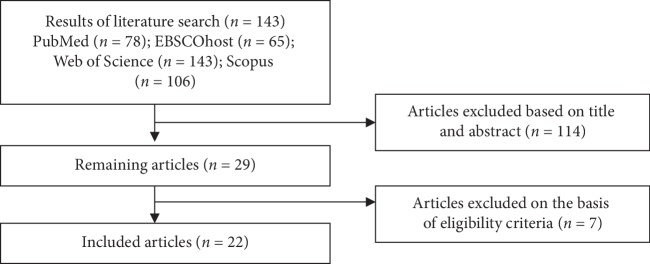
Diagram flow for the study search and selection.

The papers were checked for relevant content and were included based on the following criteria: full-text article published in English, including a comprehensive description of the shock wave application. Conference abstracts, proceedings, case reports, systematic reviews, meta-analyses, and narrative reviews were excluded. The results of the study selection procedure are summarized in a flow diagram (Figure 1).

### 2.3. Data Extraction and Quality Assessment

Three authors selected the studies and extracted their characteristics and results. All three are practising physiotherapists who apply extracorporeal shock wave therapy in patients with different musculoskeletal dysfunctions. They are also experienced in carrying out research and are fluent in English.

Table 1 presents scientific publications extracted from the Physiotherapy Evidence Database (PEDro) that had been rated with the PEDro scale. The PEDro scale (range from 1 to 10 points) is a valid measure of the methodological quality of randomized clinical trials [24]. On the basis of the PEDro score, the methodological quality was rated as high (PEDro score: 7 and more), medium (PEDro score: 4–6), or low (PEDro score: 3 or below) [25].

## 3. Results

The search and databases yielded a total of 143 articles (the majority were indexed in the Web of Science), of which only 22 met the inclusion criteria (Figure 1).

Most studies on the effectiveness of ESWT for Achilles tendinopathy included in this narrative review are randomized controlled trials [6, 17–23, 26–28]. Two case-control studies [7, 29], a case series study [30], prospective audit [31], clinical trial protocol [32], and a pilot study [33] were also considered. The majority were prospective studies [6, 7, 17, 19–23, 26–30, 32–34]. Only Erroi et al. [35], Furia [7, 29], and Wei et al. [36] presented the findings from retrospective observations.

The methodological quality of scientific publications extracted from the PEDro database was rated as high [6, 17, 20–22], or medium [18, 19, 23]. All these studies had a quality score ranging from 4 to 9 points. The study by Rasmussen et al. scored the highest, i.e., 9 of 10 points [21], while the study by Notarnicola et al. had a sum score of 4 [19].

## 4. Discussion

The likelihood of full recovery to physical activity from chronic symptoms typical of tendinopathy has been estimated at 80% [13]. ESWT is a conservative treatment that seems to yield promising response rates in patients with Achilles tendinopathy.

Lee et al. investigated the long-term outcome and factors affecting the prognosis of ESWT for chronic refractory Achilles tendinopathy [37]. Immediate treatment success was associated with an absence of a retrocalcaneal enthesophyte on X-ray, presence of pretreatment abnormal ultrasonography echogenicity, shorter mean duration of “posttreatment soreness,” and shorter duration of “posttreatment soreness after first ESWT.” The only prognostic factor associated with long-term success was the duration of “posttreatment soreness after first ESWT.”

Trials with high methodological quality ratings (PEDro scale) revealed that 4 months after completion of radial shock wave therapy (2000 pulses, 8 Hz, 2.5-3 bars, and 3 sessions), the success rate in patients suffering from noninsertional Achilles tendinopathy was 52%, while 64% of the patients with chronic insertional Achilles tendinopathy confirmed complete recovery or marked improvement [22]. An approach combining eccentric loading and radial shock wave therapy (2000 pulses, 8 Hz, 3 bars, and 3 sessions) increased the proportion of “completely recovered” or “much improved” patients to 82% [6]. It should be noted, however, that other authors did not find any beneficial effects in the shock wave therapy group compared to the control [23]. ESWT also did not prove to be superior to other therapies, including platelet-rich plasma injections [35], peritendinous hyaluronan injections [27], high-volume image-guided injection [34], Cold air and High-Energy Laser Therapy [19], or endoscopy-assisted radiofrequency ablation [36]. Rasmussen et al. [21] and Wheeler and Tattersall [34] reported significant improvements following ESWT procedures performed in patients with chronic noninsertional Achilles tendinopathy; however, only a few parameters of the patients' clinical condition and activity level were affected.

Contrasting ESWT outcomes seem to result from the complexity of Achilles tendon dysfunction, differences in shock wave application, and different methods of therapy result objectivization.  Table 2 shows the intervention characteristics and major conclusions of studies on the effectiveness of ESWT for Achilles tendinopathy.

### 4.1. Achilles Tendon Dysfunction Characteristics

A chronic Achilles tendinopathy duration of more than three months was the basic eligibility criterion in the majority of the studies [21, 30, 31]. A four-month duration was selected as the eligibility criterion by Costa et al. [23], while others considered patients who had been suffering for six months [6, 7, 17, 19, 20, 22, 28, 29, 34–36]. Santamato et al. [33] and Lynen et al. [27] used shorter eligibility periods, i.e., 4 and 6 weeks, respectively, while several authors did not specify the duration of Achilles tendinopathy symptoms at all [26, 32, 38]. Regarding acute tendinopathy, Ciccotti et al. [41] emphasized that although patients describe the pain as acute, the degenerative character of the dysfunction indicates that the tendon's adaptation to tensile overloading has been impaired long before symptom occurrence.

Timely differentiation between acute or chronic tendinopathy is essential for diagnostic and therapeutic considerations [42]. Taylor et al. believed that the effects of radial shock wave therapy are better in patients over the age of 60 and with a symptom duration of less than 12 months [31]. Older patients with longer symptom durations are less likely to benefit from ESWT [22]. Other studies failed to show a significant correlation between pain severity and functional/activity impairments [34]. The likelihood of spontaneous regeneration in patients with midportion Achilles tendinopathy of more than 6-month duration is low [22].

The ESWT outcome also depends on the location of the Achilles tendon injury and the presence of other tendon pathologies. The studies were carried out among patients with insertional [19, 20, 26, 28–30, 32, 35, 36, 38] and noninsertional Achilles tendinopathy [6, 7, 22, 27, 33]. Rasmussen [21] and Vahdatpour [17] did not specify the type of tendinopathy they had studied. A prospective audit [31] carried out in a group of 46 patients revealed that radial shock wave therapy (2500 pulses, 10 Hz, 1.5–2.5 bars, and 3 sessions) significantly reduced tendon pain and improved the performance in patients with both insertional and noninsertional refractory Achilles tendinopathy over the two-year period of therapy completion. However, an increase in patients' satisfaction at all follow-up consultations (i.e., at 6 and 16 weeks and 2 years of therapy completion) was only observed in the group with noninsertional tendinopathy [31].

Achilles tendinopathy tends to be associated with Haglund's deformity. A retrospective study by Wu et al. confirmed that the cooccurrence of these two conditions limited the effectiveness of radial shock wave therapy [38]. Haglund's deformity was among the exclusion criteria in several trials [20, 35].

### 4.2. Methods of Shock Wave Application

The two modalities of shock wave therapy used for Achilles tendinopathy are focused shock waves [7, 17, 23, 29, 33, 35] and radial shock waves [6, 17, 18, 20, 22, 30–32, 38]. The volume of tissue affected is different for each of these modalities. Most lesions treated with focused shock waves need accurate pretreatment identification using ultrasound. Maximal energy is delivered into a focused point at a predetermined tissue depth. In radial shock wave therapy, the energy is dissipated over a large area. The type of transmitter applied to the skin and the way pressure waves penetrate the tissue cause the maximal flux energy to be reached at the skin surface, not at a selected depth [43]. Njawaya et al. studied the effect of ESWT in patients suffering from calcific Achilles tendinopathy [18]. The application of 2000 pulses (15 Hz, with an energy increase from 1.4 to 1.8 bars, and sessions 3 through 5) was equally beneficial in patients with and without ultrasound navigation of the shock wave [18].

The search of the science databases did not yield any articles comparing the effects of radial and focused shock wave therapies on Achilles tendinopathy.

Vahdatpour et al. evaluated the effectiveness of a combination (radial plus focused) shock wave therapy [17]. Patients with chronic Achilles tendinopathy received four ESWT sessions (RSWT: 3000, 2.21 Hz, and 1.8–2.6 mJ/mm^2^; FSWT: 1500, 2.3 Hz, and 0.25–0.4 mJ/mm^2^) and, additionally, a 4-week physical-supportive treatment. Posttherapy American Orthopaedic Foot and Ankle Society and Visual Analog Scale scores were significantly improved compared to the sham ESWT group.

While treating Achilles tendinopathy, most researchers used 3 sessions of ESWT [6, 18–20, 22, 26, 28, 31] with a one-week break in between [6, 20, 22, 26, 31]. There were also shorter breaks of 3 to 4 days [19, 28] or longer 2-week breaks [30, 32]. The number of pulses per session ranged from 800 to 3000 [6, 18, 20, 22, 26, 30–32, 38]; the pulse frequency was 4 to 50 Hz [6, 18, 20–22, 30–32, 38]. Focused shock waves were applied once [7, 29], three [23, 35] or five times [33], once a week [33, 35], or once a month [23]. The number of pulses was 1500 to 3000 [7, 23, 29, 33, 35]. However, only Santamato et al. [33] and Furia [7, 29] specified the pulse frequency, which was 1–4 Hz.

For neuromuscular dysfunction, the effect of shock wave therapy tends to be dose-dependent and causes symptom improvement over time [44]. The intensity of radial shock waves applied for Achilles tendinopathy was 1.4–3 bars [6, 18, 20, 22, 26, 31, 32], whereas in the case of focused shock wave therapy, the energy flux density was 0.12–0.4 mJ/mm^2^ [7, 17, 23, 29, 33, 35].

Low-energy extracorporeal shock wave therapy is usually quite well tolerated. Since discomfort experienced during impulse application is rather mild, there is no need for anaesthesia, and the treatments can be repeated. High-energy ESWT tends to be more painful and therefore requires local analgesics. However, pain assessment at 12 months after a single application of high-energy extracorporeal shock wave therapy for insertional Achilles tendinopathy (3000 pulses and total energy flux density of 604 mJ/mm^2^) revealed that the pain-alleviating effects of this procedure were significantly better in the patients who received nonlocal anaesthesia compared to those who had been treated using a local anaesthesia field block [29].

Taylor et al. emphasized the risks of high-dose ESWT and therefore did not use it in patients with refractory Achilles tendinopathy [31]. Chao et al. demonstrated that a low-energy level with a low number of impulses (0.36 mJ/mm^2^ with 50 and 100 impulses) had positive stimulatory effects, while a high-energy level and high number of impulses (0.68 mJ/mm^2^ with 250 and 500 impulses) had significant inhibitory effects [45].

A considerable majority of researchers did not observe any significant adverse effects during or after extracorporeal shock wave application. Several authors reported transient posttherapy skin reddening but no bruising [6, 20, 22, 31, 35, 38]. Some patients only mentioned mild to moderate discomfort [31]. Costa et al. reported Achilles tendon rupture within two weeks of the first shock wave therapy session [23]. Although they did not consider this to be an adverse effect of focused shock waves, they strongly suggested carrying out meticulous diagnostic tests prior to ESWT application, especially in patients over the age of 60 years [23].

Shock wave treatment can be combined with other therapies. Several authors suggested that a combination of ESWT and eccentric exercises was an effective treatment for insertional Achilles tendinopathy [6, 30, 32]. The trial by Rompe et al., which scored high on the PEDro scale (Table 1), compared the effectiveness of two management strategies for midportion Achilles tendinopathy [6]. At the 4-month follow-up, eccentric loading alone was less effective with respect to pain alleviation and function improvement than a combination of eccentric loading and repetitive ESWT. The authors emphasized that 82% of the combined regimen group reported complete recovery or significant improvement.

Notarnicola et al. showed greater improvement in clinical and functional conditions as well as reduced tendon perfusion following a combination of ESWT with daily dietary supplementation (arginine-L-alpha-ketoglutarate and hydrolysed collagen type I) compared to ESWT alone [28].

### 4.3. Evaluation of Therapy Outcomes

The majority of researchers used subjective methods to evaluate ESWT outcomes in patients with Achilles tendinopathy [6, 7, 17–23, 26, 27, 29–31, 33–35, 38].

The most frequently used subjective measures was the Visual Analog Scale (VAS) for pain [7, 17–19, 21, 23, 26, 27, 29–36] and the Victorian Institute of Sport Assessment-Achilles questionnaire (VISA-A) [6, 18, 20, 22, 26, 27, 32–36, 38]. The VISA-A questionnaire is a valid and reliable index of the clinical severity of Achilles tendinopathy. It should be noted that the questionnaire is not a diagnostic tool, and cooccurrence of other conditions that might affect the lower limb function reduces the VISA-A scores [39].

Other instruments measuring treatment outcomes, such as the American Orthopaedic Foot and Ankle Society (AOFAS) score [17, 21, 30, 32, 36, 40], Roles and Maudsley score [7, 19, 29, 33, 35], and 6-point Likert satisfaction score [6, 20, 22, 31, 38], have also been used.

Objective measurements were also employed, including ultrasound examination of the anteroposterior diameter of the Achilles tendon [6, 22], power Doppler ultrasonography to evaluate the vascularization of the affected Achilles tendons [27, 33], oximetry [28], and digital pressure algometry [32].

Cheng et al. investigated the utility of ultrasonography for the assessment of ESWT effectiveness in patients with insertional Achilles tendinopathy [46]. Ultrasound imaging facilitated the evaluation of changes in the thickness and crosssectional area of the Achilles tendon, size of calcific plaques, tendon structure, and neovascularization. Nevertheless, the authors concluded that the outcome of ESWT in insertional Achilles tendinopathy cannot be predicted by the variables observed by ultrasonography [46].

Literature reviews clearly illustrate the need for clinical trials in which the outcomes of ESWT for Achilles tendinopathy will be objectively measured. The accuracy and reliability of such objective measurements should also be determined.

### 4.4. Mechanisms of Shock Wave Action

The mechanisms of ESWT action on human tendinous tissue seem complex and remain to be fully elucidated [47].

Waugh et al. proposed a hypothesis that shock waves might promote proinflammatory and catabolic processes associated with the removal of damaged matrix constituents [48]. Repair processes promoted by extracorporeal shock waves have been attributed to tenocyte proliferation and collagen synthesis [45, 49]. It has also been speculated that shock waves might reactivate the healing process through microdisruption of avascular or minimally vascularized tissues [50], resulting in neovascularization, improved blood supply, and stimulation of tissue regeneration [51].

Numerous researchers have observed pain relief by extracorporeal shock wave application [6, 17, 19, 22, 26, 33, 35, 40]. Shock wave-related pain relief has been attributed to a decrease in substance P release from the treated area [52], selected loss of unmyelinated nerve fibres at the sites of shock wave application [53], activation of the serotonergic system [54], or pain suppression system at the level of the spinal cord [55].

Optimization of collagen synthesis, maturation, and strength progressively increases tendon tensile strength and hence recovery [13]. Collagen neosynthesis [13] and neovascularization [44] within the affected tendon seem to account for the gradual and long-term benefits of shock wave therapy in tendinopathy.

It should be noted that ESWT effectiveness was not assessed immediately after the completion of extracorporeal shock wave therapy but several weeks or months later. Rompe et al. suggested that collagen turnover and remodelling needed time, and hence the main follow-up should be carried out no sooner than 4 months from baseline [22]. Shock wave therapy does not induce rapid symptom improvement; rather, it initiates reparative processes within injured tissues [56]. Patients with Achilles tendinopathy experienced gradual pain relief and functional capacity improvement [7, 19, 26, 27, 29, 35, 40].

## 5. Conclusions

The complexity of the biological response to shock waves, the high diversity of application methodologies, and the lack of objective measurements all prevent ESWT effectiveness for Achilles tendinopathy from being fully determined. There are knowledge gaps yet to be researched, and the results of experimental studies remain contradictory. Our literature review shows that irrespective of the ultimate outcomes, shock wave therapy is a safe, well-tolerated treatment modality.

Nevertheless, there is a need for further multidirectional and multicentre, randomized controlled studies on the effectiveness of shock waves for Achilles tendinopathy that should fulfil the criteria for evidence-based medicine.

## Figures and Tables

**Table 1:** Scientific publications extracted from the Physiotherapy Evidence Database (PEDro) that had been rated with the PEDro scale.

Reference	Eligibility criteria specified	Subjects randomly allocated to groups	Allocation concealed	Groups similar at baseline	Blinding of all subjects	Blinding of all therapists	Blinding assessors	>85% follow-up	Intention-to-treat analysis	Between-group statistical comparison	Point and variability measures	Score^*∗*^
Rompe et al. [6]	+	+	+	+			+	+	+	+	+	8
Vahdatpour et al. [17]	+	+		+	+		+	+	+	+	+	8
Njawaya et al. [18]	+	+	+	+				+		+		5
Notarnicola et al. [19]	+	+		+						+	+	4
Rompe et al. [20]	+	+	+	+			+	+	+	+	+	8
Rasmussen et al. [21]	**+**	**+**	**+**	**+**	**+**		**+**	**+**	**+**	**+**	**+**	9
Rompe et al. [22]	+	+	+	+			+	+	+	+	+	8
Costa et al. [23]	+	+	+				+	+	-	+	+	6

^*∗*^ *Eligibility criteria* item is not included in PEDro score calculations.

**Table 2:** Studies on the effectiveness of extracorporeal shock wave therapy for Achilles tendinopathy.

Reference	Sample size	Groups	Duration of symptoms	Type of shock wave therapy	Number of shocks/frequency	Energy flux density	Number of sessions	Outcome measure	Follow-up	Study conclusions
Rompe et al. [6]	68	I: eccentric loading training II: eccentric loading training + ESWT	>6 months	RSWT	2000; 8 Hz	3 bars; 0.1 mJ/mm^2^	3 sessions, once a week	VISA-A; general assessment by 6-point Likert scale; 11-point NRS; anteroposterior diameter of Achilles tendon of affected and unaffected leg	6 and 16 weeks	At the 4-month follow-up, for all outcome measures, the ESWT + eccentric loading training group showed significantly more favorable results than the group I
Furia [7]	68	I: ESWT II: control group	>6 months	FSWT	3000 shocks; 1–4 Hz	0.21 mJ/mm^2^	1 session	VAS; Roles and Maudsley score	1, 3, and 12 months	ESWT group exhibited better therapy outcomes compared to control participants
Vahdatpour et al. [17]	43	I: ESWT II: sham ESWT	>6 months	Combination of RSWT + FSWT in one session	RSWT: 3000; 2.21 Hz FSWT: 1500; 2.3 Hz	RSWT: 1.8–2.6 mJ/mm^2^ ESWT: 0.25–0.4 mJ/mm^2^	4 sessions, once a week	AOFAS; VAS	Immediately after the end of treatment, 4 and 16 weeks	Mean AOFAS and VAS scores differed significantly between ESWT and sham ESWT groups at 16 weeks of therapy completion
Maffulli et al. [26]	80	I: ESWT	Not known	RSWT	500 + 2500	1.5 + 2.5 bars	3 sessions, once a week	VAS; VISA-A; EuroQolt 5D	3, 6, 12, and 24 months	Significant improvement of VAS, EQ-5D mobility, EQ-5D pain/discomfort, and EQ-5D usual activities scores at 3 months of therapy completion. VISA-A improvement at 12 months of therapy completion
Njawaya et al. [18]	31	I: patient-guided ESWT II: ultrasound guided	Effect of ultrasound evaluated irrespective of symptom duration	RSWT	2000; 15 Hz	1.4–1.8 bars	3 to 5 sessions	VAS; VISA-A	6 weeks, 3 and 6 months	No difference between group I and II results in terms of pain or function outcome at 3 or 6 months of follow-up
Lynen et al. [27]	59	I: hyaluronan injections II: ESWT	>6 weeks	Not known	1500; 4 Hz	Not known	3 sessions, once a week	VAS; VISA-A; CGI; power Doppler ultrasonography; the intensity of clinical parameters	4 weeks, 3 and 6 months	Two hyaluronan injections yielded greater treatment success in Achilles tendinopathy than standard ESWT
Notarnicola et al. [19]	60	I: CHELT therapy (Cold air and High-Energy Laser Therapy) II: ESWT	>6 months	Not known	1600	0.05–0.07 mJ/mm^2^	3 sessions at 3- to 4-day intervals	VAS; ankle-hindfoot scale; Roles and Maudsley score	Immediately after the end of treatment, 2 and 6 months	Compared to ESWT, CHELT therapy more effectively reduces pain and improves function in patients with insertional Achilles tendinopathy
Notarnicola et al. [28]	64	I: ESWT + dietary supplements II: ESWT + placebo (control group)	>6 months	Not known	1600	0.05–0.07 mJ/mm^2^	At 3- to 4-day intervals	VAS; ankle-hindfoot scale; Roles and Maudsley score; oximetry	2 and 6 months	Patients with insertional Achilles tendinopathy showed greater improvement in clinical and functional condition as well as reduced tendon perfusion following a combination of ESWT dietary supplements compared to ESWT alone
Rompe et al. [20]	50	I: eccentric loading training II: ESWT	Over 6 months	RSWT	2000; 8 Hz	2.5 bars; 0.12 mJ/mm^2^	3 sessions, once a week	VISA-A; general assessment by 6-point Likert scale; 11-point numeric rating scale (NRS); pain threshold; tenderness at 3 kg assessed on a NRS from 0 to 10	6 and 16 weeks	For all outcome measures, the ESWT group showed significantly more favorable results than group I with eccentric loading only
Rasmussen et al. [21]	48	I: ESWT II: sham ESWT	>3 months	RSWT	ESWT: 2000; 50 Hz Sham ESWT: 2000; 50 Hz	ESWT: 0.12–0.51 mJ/mm^2^ Sham ESWT: 0 mJ/mm^2^	4 sessions, once a week	AOFAS score; VAS	4, 8, and 12 weeks	ESWT resulted in functional improvement but did not have a clear impact on pain severity
Rompe et al. [22]	75	I: eccentric loading training II: low-energy ESWT III: wait-and-see policy	>6 months	RSWT	2000; 8 Hz	3 bars; 0.1 mJ/mm^2^	3 sessions, once a week	VISA-A; general assessment by 6-point Likert scale; 11-point numeric rating scale (NRS); pain threshold; tenderness; anteroposterior diameter of Achilles tendon of affected and unaffected leg	6 and 16 weeks	For all outcome measures, group I (eccentric loading) and II (ESWT) showed significantly better results than group III (wait-and-see policy)
Costa et al. [23]	49	I: ESWT II: placebo	>4 months	FSWT	1500; not known	Max 0.2 mJ/mm^2^	3 sessions, once a month	VAS; ranges of motion at the ankle joint; calf diameter; tendon diameter; single-leg heel rise; single-leg tiptoe jump; FIL; EuroQol generalized health status questionnaire	3 months, 1 year	There was no difference between the groups in pain relief, range of motion at the ankle or differences in the FIL or EQol scores
Furia [29]	68	I: ESWT Ia: local anaesthesia field block Ib: nonlocal anaesthesia II: control group	>6 months	FSWT	3000 shocks; 1–4 Hz	0.21 mJ/mm^2^	1 session	VAS; Roles and Maudsley score	1, 3, and 12 months	ESWT proved an effective treatment for chronic insertional Achilles tendinopathy. Local field block anaesthesia may decrease the effectiveness of this procedure
Pavone et al. [30]	40	I: ESWT + eccentric exercises	>3 months	RSWT	800; 4 Hz	14 keV	4 sessions, with a 2-week interval	VAS; AOFAS hindfoot score	2, 6, and 12 months	ESWT combined with eccentric exercises proved effective in patients with chronic insertional Achilles tendinopathy
Taylor et al. [31]	46	I: insertional Achilles tendinopathy II: noninsertional Achilles tendinopathy	>3 months	RSWT	2500; 10 Hz	1.5 bars–2.5 bars	3 sessions, once a week	VAS at rest and on activity; VISA-A; Likert satisfaction score	6 and 16 weeks, 2 years	ESWT appeared to be beneficial in the long-term improvement of pain and functional outcome in patients with refractory insertional and noninsertional Achilles tendinopathy
Mansur et al. [32]	19	I: ESWT + eccentric exercises	Not known	RSWT	2000–3000; 7–10 Hz	1.5–2.5 bars	2 sessions, once every two weeks	VAS; VISA-A; AOFAS; algometry	24 weeks	Eccentric exercises combined with ESWT significantly improved patients' symptoms
Santamato et al. [33]	12	I: ESWT	>4 weeks	FSWT	1600; 4 Hz	0.12 mJ/mm^2^	5 sessions, once a week	VAS; VISA-A; range of motion active dorsiflexion and plantar flexion ankle; Roles and Maudsley score	1 and 3 months	ESWT significantly improved clinical condition but did not cause neovascularization
Wheeler and Tattersall [34]	63	I: ESWT II: high-volume image-guided injection	Not known	Not known	2000; 10 Hz	2.1 ± 0.3–2.9 ± 0.4	3 sessions, once a week	VAS; VISA-A; MOXFQ	6 weeks, 3 and 6 months	Statistically significant improvement in groups I and II. No significant intergroup differences
Erroi et al. [35]	45	I: ESWT II: platelet-rich plasma	>6 months	FSWT	2400; not known	0.17–0.25 mJ/mm^2^	3 sessions, once a week	VISA-A; VAS; Roles and Maudsley score	Immediately after the end of treatment, 2, 4, and 6 months	Both therapeutic modalities are safe and effective
Wei et al. [36]	78	I: endoscopy-assisted radiofrequency ablation II: ESWT III: eccentric exercises	>6 months	Not known	2000; 10 Hz	0.12 mJ/mm^2^	3 sessions, once a week	AOFAS; VAS; VISA-A	6, 12, and 18 months	Endoscopy-assisted radiofrequency ablation yielded better outcome than ESWT
Wu et al. [38]	67	I: with Haglund's deformity II: without Haglund's deformity	Not known	RSWT	2000; 8 Hz	0.12 mJ/mm^2^	5 sessions, once a week	VISA-A score; 6-point Likert scale		Haglund's deformity adversely affected ESWT outcome
Carulli et al. [40]	102	I: ESWT	>3 months	Not known	2400	0.08–0.33 mJ/mm^2^	3 sessions, once a month	NRS; AOFAS	1, 6, and 12 months	ESWT reduces pain and improves function in patients with chronic Achilles tendinopathy

ESWT, extracorporeal shock wave therapy; FSWT, focused shock wave therapy; RSWT, radial shock wave therapy; VAS, Visual Analog Scale; VISA-A, Victorian Institute of Sport Assessment-Achilles questionnaire; AOFAS, American Orthopaedic Foot and Ankle Society score; NRS, numeric rating scale; FIL, Functional Index of Lower Limb Activity; CGI, Clinical Global Impression; MOXFQ, Manchester-Oxford Foot Questionnaire.
